# Nerve Repair and Orthodromic and Antidromic Nerve Grafts: An Experimental Comparative Study in Rabbit

**DOI:** 10.1155/2020/5046832

**Published:** 2020-01-02

**Authors:** Jihyeung Kim, Young Eun Choi, Jeong Hwan Kim, Seung Hak Lee, Sohee Oh, Sae Hoon Kim

**Affiliations:** ^1^Department of Orthopedic Surgery, Seoul National University College of Medicine, Seoul National University Hospital, Seoul, Republic of Korea; ^2^Department of Orthopedic Surgery, Seoul Medical Center, Seoul, Republic of Korea; ^3^Department of Rehabilitation Medicine, Asan Medical Center, College of Medicinel, University of Ulsan, Seoul, Republic of Korea; ^4^Medical Research Collaborating Center, Seoul National University, Boramae Medical Center, Seoul, Republic of Korea

## Abstract

**Purpose:**

Although many surgeons have anecdotally described reversing the polarity of the autograft with the intent of improving regeneration, the optimal orientation of the autogenous nerve graft remains controversial. The aim of this study was to compare (1) the outcomes of orthodromic and antidromic nerve grafts to clarify the effect of nerve graft polarity and (2) the outcome of either form of nerve grafts with that of nerve repair.

**Methods:**

In 14 of the 26 rabbits used in this study, a 1 cm defect was made in the tibial nerve. An orthodromic nerve graft on one side and an antidromic nerve graft on the other were performed using a 1.2 cm long segment of the peroneal nerve. In the remaining 12 rabbits, the tibial nerve was transected completely and then repaired microscopically on one side but left untreated on the other. Electrophysiologic studies were performed in all animals at 8 weeks after surgery, and the sciatic nerves were harvested.

**Results:**

Compound motor action potential was visible in all rabbits treated by nerve repair but in only half of the rabbits treated by nerve graft. There was no significant difference in the compound motor action potential, nerve conduction velocity, or total number of axons between the orthodromic and antidromic nerve graft groups. However, in both groups, the outcome was significantly poorer than that of the nerve repair group.

**Conclusion:**

There was no significant difference by electromyographic or histologic evaluation between orthodromic and antidromic nerve grafts. Direct nerve repair with moderate tension may be a more effective treatment than nerve grafting.

## 1. Introduction

When surgery is required to repair a transected nerve or when a nerve injury requires excision, the best outcomes are achieved by direct nerve repair without grafting [1]. However, if there is a break in the continuity of the nerve such that the gap cannot be bridged without tension, an autogenous nerve graft is usually indicated. Although autogenous nerve grafting is the gold standard for bridging the gap, it has several disadvantages such as an increased operative time, need for additional surgical incisions, donor site morbidity, low donor nerve availability, and diameter mismatch [2]. To restore motor and sensory functions after autogenous nerve graft, the regenerated axons should grow into and pass through the graft, finally reaching the distal end of the defective nerve [3]. The outcomes of autogenous nerve graft will be inferior to those of nerve repair; however, studies comparing the outcomes of the two approaches are lacking.

According to anecdotal reports, many surgeons reverse the polarity of the autograft during autogenous nerve grafting, with the intent of improving nerve regeneration by mitigating the potential misrouting effects of arborization [4]. However, the optimal orientation of an autogenous nerve graft remains controversial. In a 1943 study, Sanders and Young [5] found no significant difference in outgrowth distance between grafts in which the polarity was reversed and those in which it remained unchanged. Stromberg et al. also compared nerve graft polarity in rats using a 1 cm segment of the sciatic nerve. They concluded that the function of the nerve graft was independent of its polarity [6]. In the study of Nakatsuka et al., which evaluated the effect of cable nerve graft polarity, there was no significant difference in either motor conduction velocity or muscle weight as a functional outcomes of nerve graft orientation [7]. However, Ansselin and Davey [8] concluded that regeneration of axons to their peripheral targets is facilitated by reversing the graft orientation. This was disputed by Millesi, who found that nerve graft reversal did not enhance regeneration [9]. In a systematic review of the effect of autograft polarity on functional outcomes following peripheral nerve repair surgery, it was concluded that there were insufficient data suggesting that the polarity of an autologous nerve graft impacts on nerve regeneration and functional outcome [4]. Furthermore, only six studies were included in that review, and most of them were conducted more than 20 years ago.

Several authors have used animal models to evaluate regeneration of the peripheral nerve [5, 7, 10–12]. Sanders and Young [5] used rabbit peroneal nerve, Nakatsuka et al. [7] used common peroneal nerve, and Whitworth et al. [10] used sciatic nerve. In the present study, we compared the outcomes of orthodromic and antidromic nerve graft to clarify the effect of nerve graft polarity on nerve regeneration in a rabbit model. We then compared those outcomes to that of nerve repair.

## 2. Materials and Methods

Twenty-six mature New Zealand White male rabbits (mean age, 24 weeks; weight, 3.5–4.0 kg) were used in this study. Of these, fourteen rabbits were allocated to the nerve-graft groups. Orthodromic nerve grafting was performed on one hindlimb, and antidromic nerve grafting was performed on the other hindlimb. The remaining twelve rabbits were allocated to the nerve repair and control groups. Nerve repair was performed on one side, and no surgical procedure was performed on the contralateral side. All surgical experiments were conducted with approval from the Experimental Animal Committee of the Clinical Research Institute of our institute (IACUC No.: 17-0198). The animals were housed individually with a 12 h light-dark cycle, and food and water were provided *ad libitum*. Prior to the surgical procedures, the rabbits were anesthetized with intramuscular zoletil (10 mg/kg body weight) and xylazine (10 mg/kg body weight). After surgery, pain was managed with meloxicam (0.2 mg/kg body weight).

### 2.1. Nerve Grafting

**Figure 1:**
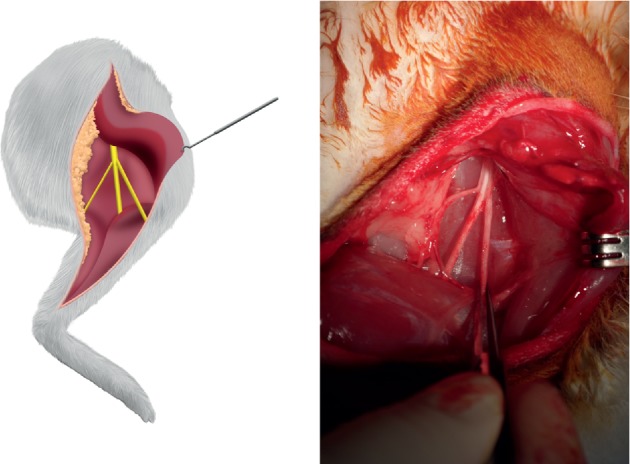
The sciatic nerve and its trifurcation were exposed through a posterolateral approach on the hindlimb.

**Figure 2:**
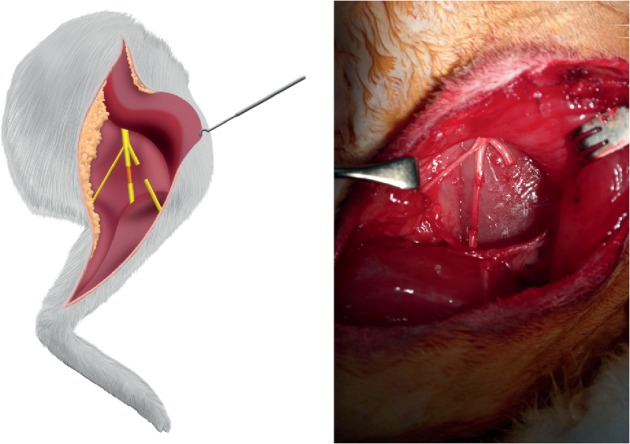
A tibial nerve defect was reconstructed using a peroneal nerve segment positioned orthodromically on one side and antidromically on the other.

The sciatic nerve was approached through a posterolateral longitudinal skin incision centered on the knee joint and then exposed in the interval between the gluteus superficialis and biceps femoris muscles (Figure 1). After identification of the trifurcation site of the sciatic nerve, where it separates into its tibial, peroneal, and sural branches, the tibial nerve was transected completely 1 and 2 cm proximal to the point where it entered the gastrocnemius muscle. A 1.2 cm long nerve segment was then harvested from the peroneal nerve beginning 1 cm distal to the trifurcation and extending distally. The surgical procedures used in the two hindlimbs were identical. The harvested nerve segment was grafted orthodromically on one hindlimb and antidromically on the other (Figure 2). The nerve grafts were sutured microscopically using 10-0 nylon sutures.

### 2.2. Nerve Repair

**Figure 3:**
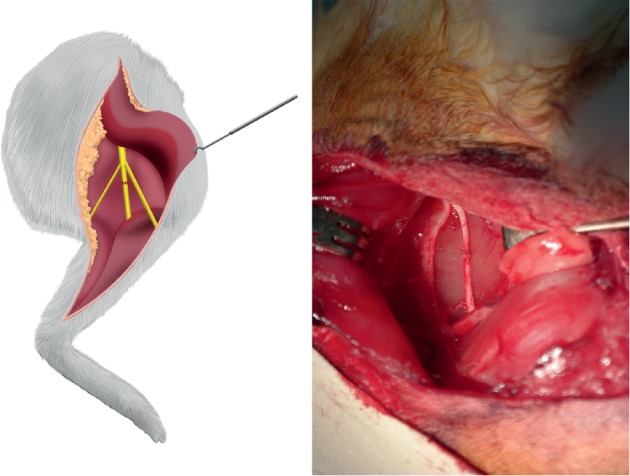
The tibial nerve was transected completely and then repaired microscopically.

The same approach was used to identify the sciatic nerve and its trifurcation in one hindlimb. The tibial nerve was transected completely 2 cm proximal to the point where it entered the gastrocnemius muscle and then repaired microscopically using 10-0 nylon sutures (Figure 3). No surgical procedure was performed on the contralateral hindlimb.

**Figure 4:**
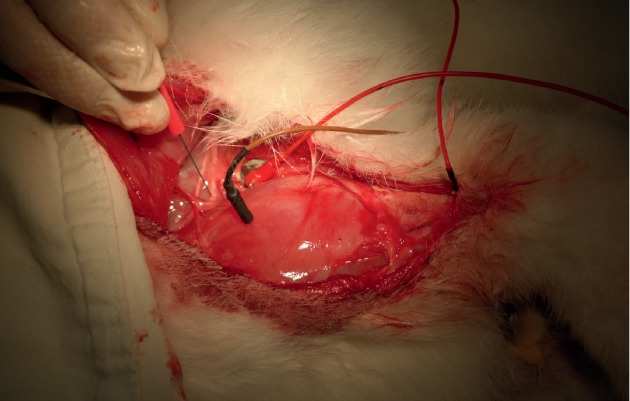
The outcome of nerve regeneration was evaluated eletromyographically. The tibial nerve was stimulated with a needle electrode, and the compound muscle action potential (CMAP) was recorded.

Eight weeks after surgery, all animals were again anesthetized and the sciatic nerve was exposed using the same approach. The outcome of reinnervation of the gastrocnemius was evaluated by electromyography (EMG) and nerve conduction studies (NCSs) using a 2-channel portable electrodiagnostic system (Medelec Synergy Plinth; Oxford Instruments, UK) (Figure 4). The compound motor action potential (CMAP) of the gastrocnemius muscles was measured using needle electrodes. Supramaximal nerve stimulation was conducted using a custom-made bipolar stimulator also equipped with needle electrodes (scalp needle electrode; Natus Alpine Biomed, Denmark), with the interelectrode distance fixed at 0.5 cm. Nerve stimulations were performed both proximal and distal to the nerve graft, and the distance between the stimulations was measured to calculate the nerve conduction velocity.

After the NCS and EMG studies, all animals were fully anesthetized and euthanized with carbon dioxide. The tibial nerve was harvested 5 mm proximal to the point where it entered the gastrocnemius muscle. The nerve specimens were fixed in neutral buffered 10% formalin (pH 7.4), and paraffin blocks were made. Thin sections (5 *μ*m) were cut in the transverse plane, mounted, and stained for immunohistochemistry as follows. The sections (5 *μ*m) were dewaxed in a standard xylene wash followed by rehydration. Antigen retrieval was performed by placing the sections in epitope retrieval solution (0.01 M citrate buffer, pH 6.0) and then microwaving them for 20 min, after which they were incubated in 3% hydrogen peroxide for 20 min at room temperature and blocked with 10% normal goat serum for 1 h. Serial antibody staining was performed with S-100 (orb18264, 1 : 200; Biorbyt, UK) primary antibody and mouse Alexa Fluor 488 goat anti-mouse secondary antibody, followed by neurofilament (NF) (ab3966, 1 : 100; Abcam, UK) primary antibody and Alexa Fluor 488 anti-mouse secondary antibody for 1 h at room temperature. The nuclei were stained by incubating the sections for 10 min with DAPI. The number of regenerated axons was counted, and the outcomes of the three groups (nerve repair, orthodromic nerve graft, and antidromic nerve graft) were compared.

To assess significant differences in proximal CMAP, distal CMAP, and nerve conduction velocity and total axon count among orthodromic nerve graft, antidromic nerve graft, nerve repair, and control groups, a generalized estimating equation (GEE) [13,14] was computed to account for potential correlation of observations within the same rabbit. In addition, post hoc pairwise comparisons between orthodromic versus antidromic nerve graft, nerve repair versus control, orthodromic nerve graft versus nerve repair, and antidromic nerve graft versus nerve repair were conducted using the GEE. For multiple comparisons, the Bonferroni correction was used to adjust the significance level, resulting in an *α* value of 0.05/6 = 0.0083.

## 3. Results

**Figure 5:**
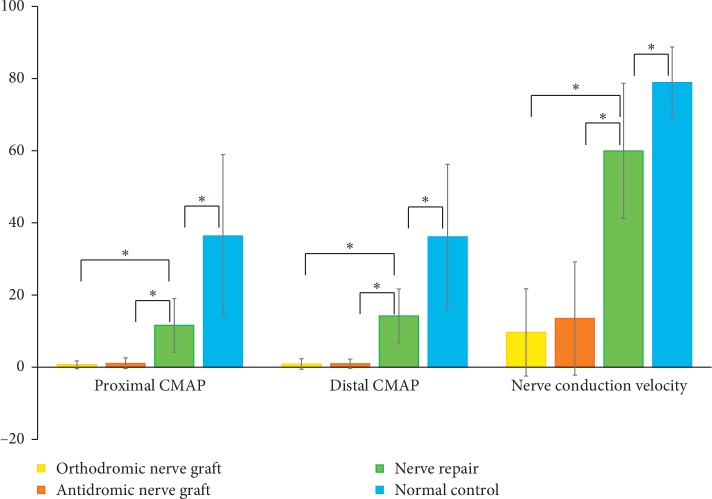
Comparisons of the proximal CMAP, distal CMAP, and nerve conduction velocity among the orthodromic nerve graft, antidromic nerve graft, and nerve repair groups. The asterisk indicates significant difference.

All rabbits survived until they were euthanized at 8 weeks after the first surgery. However, in two of the rabbits that underwent nerve grafts, bilateral ulceration and discoloration of multiple toes were seen. Of the 14 rabbits treated by nerve graft, a CMAP was visible in 6 in the orthodromic nerve graft group and 7 in the antidromic nerve graft group. A CMAP was visible in 12 rabbits treated by nerve repair. The average proximal CMAP area was 0.66 ms·mV (range: 0–3.5 ms·mV) in the orthodromic nerve graft group and 1.1 ms·mV (range: 0–4.8 ms·mV) in the antidromic nerve graft group. In the nerve repair and control groups, the corresponding values were 11.59 ms·mV (range: 2.7–25.2 ms·mV) and 36.53 ms·mV (range: 11.8–65.8 ms·mV), respectively. The average distal CMAP area was 0.88 ms·mV (range: 0–4.6 ms·mV) in the orthodromic nerve graft group and 0.95 ms·mV (range: 0–4.1 ms·mV) in the antidromic nerve graft group. In the nerve repair and control groups, the values were 14.18 ms·mV (range: 2.7–28.6 ms·mV) and 36.25 ms·mV (range: 12.2∼60.5 ms·mV), respectively. The average conduction velocity in the orthodromic and antidromic nerve graft groups was 9.64 m/s (range: 0–28.2 m/s) and 13.48 m/s (range: 0–40 m/s), respectively, compared with 59.9 m/s (range: 25.5–95 m/s) and 78.83 m/s (range: 68.2∼100 m/s) in the nerve repair and control groups. The differences in the proximal CMAP, distal CMAP, and nerve conduction velocity between the orthodromic nerve graft and nerve repair groups and between the antidromic nerve graft and nerve repair groups were significant (all *p* < 0.001) (Figure 5), as were the differences in the proximal CMAP, distal CMAP, and nerve conduction velocity between the nerve repair and control group (*p* < 0.001, *p*=0.001, and *p*=0.002, respectively). By contrast, there were no significant differences in the proximal CMAP, distal CMAP, or nerve conduction velocity between the orthodromic and antidromic nerve graft groups (*p*=0.164, *p*=0.803, and *p*=0.366, respectively) (Figure 5).

**Figure 6:**
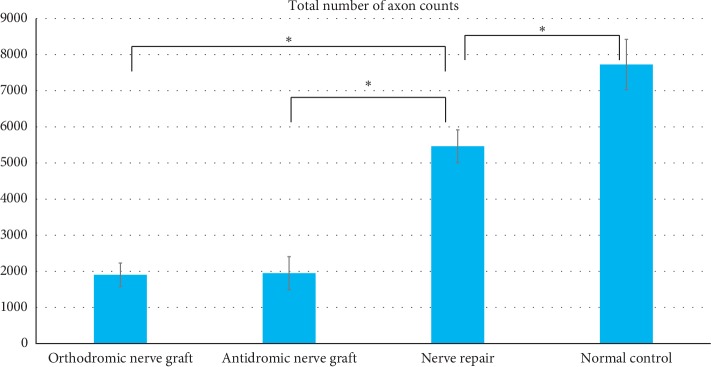
Comparison of total number of axons between the orthodromic nerve graft, antidromic nerve graft, and nerve repair groups. The asterisk indicates significant difference.

**Figure 7:**
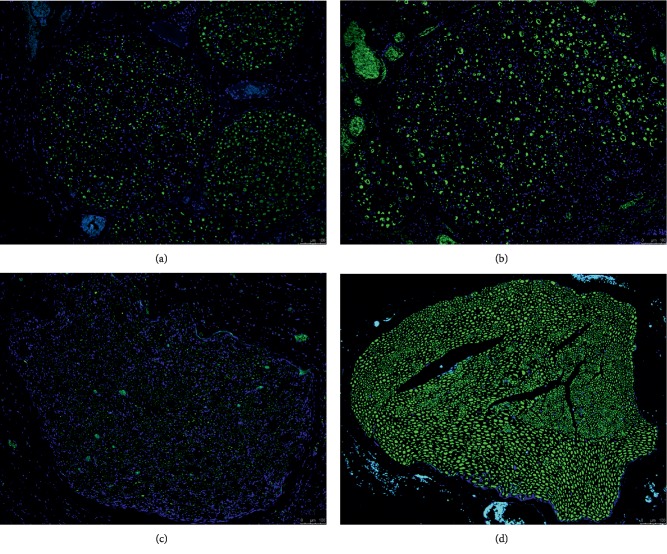
Light micrographs of neurofilament- (NF-) immunostained sections of the tibial nerve. Transverse sections of the tibial nerve 5 mm proximal to its entry site in the gastrocnemius muscle were prepared. Although the average total axon count in the nerve repair group (c) was significantly lower than in the control group (d), it was significantly greater than in the orthodromic (a) or antidromic (b) nerve graft groups. Magnification, 10×.

In the control group, the average diameter of the tibial nerve 5 mm proximal to where it entered the gastrocnemius muscle was 1.88 mm (range: 1.76–2.03 mm) and that of the peroneal nerve 1.6 cm distal to the trifurcation of the sciatic nerve was 1.35 mm (range: 1.23–1.57 mm). The total axon counts of the tibial nerve 5 mm proximal to its entry site in the gastrocnemius muscle was 1,902 (range: 1,368∼2,407) in the orthodromic nerve graft group, 1,949 (range: 1,304–2,911) in the antidromic nerve graft group (Figures 6 and 7), 5,464 (range: 4,687∼6,100) in the nerve repair group, and 7,726 (range: 6,382∼8,726) in the control group. The differences in axon count between the orthodromic nerve graft and nerve repair group and between the antidromic nerve graft and nerve repair groups were significant (all *p* < 0.001). The number of axons also significantly differed between the nerve repair and control groups (*p* < 0.001), but not between the orthodromic and antidromic nerve graft groups (*p*=0.660).

## 4. Discussion

Schmitz and Beer [15] suggested using a caudal and lateral approach to the rabbit peroneal nerve to evaluate its degeneration and regeneration. However, we used a posterolateral approach to expose the sciatic nerve and its trifurcation. Through this approach, harvesting the common peroneal nerve, grafting to the tibial nerve, and neurorrhaphy of the tibial nerve were all possible. In the present study, the peroneal nerve rather than the sural nerve was harvested, because the latter is too small for nerve grafting. Furthermore, a better outcome can be expected using a mixed, rather than pure sensory, nerve as the donor nerve. Several authors suggested that sensory nerve isografts are inferior to motor and mixed nerve isografts for the repair of a mixed nerve defect in rats [16–18]. The length of the peroneal nerve segment harvested for the repair of a 1 cm long tibial nerve defect was 1.2 cm, because a harvested nerve graft shrinks in length by ∼20% [19]. To control for the regeneration of a repaired or reconstructed nerve in the different groups, the tibial nerve was transected completely 1 cm and 2 cm proximal to its entry site in the gastrocnemius muscle in the nerve graft groups and 2 cm proximal to the same point in the nerve repair group. Because in a previous study 95% of the animals treated by nerve grafting showed signs of return of function at 8 weeks after surgery [20], this time point was chosen for the electromyographic and histological evaluations of the graft and repair procedures.

In this study, the outcome achieved with the nerve grafts was poorer than that obtained with nerve repair. A CMAP was visible in only half of the rabbits in the nerve graft groups but in all of the rabbits in the nerve repair group. In addition, even in rabbits in the nerve graft group with a detectable CMAP, it was significantly less than that in the nerve repair group. This can be explained by the fact that, during nerve repair or nerve graft, there is an unavoidable size and fascicle mismatch, as well as scarring and fibrosis from sutures, tissue handling, and the injury itself, all which can lead to poor regeneration [21]. A clinical rule of thumb is a 50% loss of axons at each coaptation site. Therefore, for primary nerve repair, ∼50% of the original axons will successfully regenerate through the repair site, while for a nerve graft with two coaptation sites, 25% of the axons will successfully regenerate through the graft. Furthermore, there is usually a size mismatch between donor and recipient nerves. Ideally, the diameter of a nerve graft should correlate exactly with those of the proximal and distal ends of the prepared host nerve [19]. However, in this study, the nerve diameter ratio of the donor peroneal nerve segment and recipient tibial nerve was 0.72 (1.35 mm/1.88 mm), corresponding to a potential cross-sectional area ratio between the two nerves of 0.52 (0.72^2^). Therefore, considering a 50% regeneration rate in the proximal and distal repair sites and the cross-sectional area ratio of the two nerves, the estimated success rate is only 13%. In a comparative study of primary repair, delayed repair, and nerve graft, good to excellent results were achieved in 78% of the primary repair group but only 33.3% of the nerve graft group [22]. Similarly, in another study comparing microsuture, interpositional nerve graft, and laser solder weld repair of the rat inferior alveolar nerve, interpositional nerve graft was the least effective [23]. If direct nerve repair is impossible or excessive tension is applied to the repaired nerve such that nerve regeneration is interrupted, a nerve graft should be performed, while taking into account the likelihood of a much poorer outcome than is the case in nerve repair. Matsuzaki et al. even suggested distal nerve elongation as an alternative to nerve grafting for the repair of large nerve defects [24].

**Figure 8:**
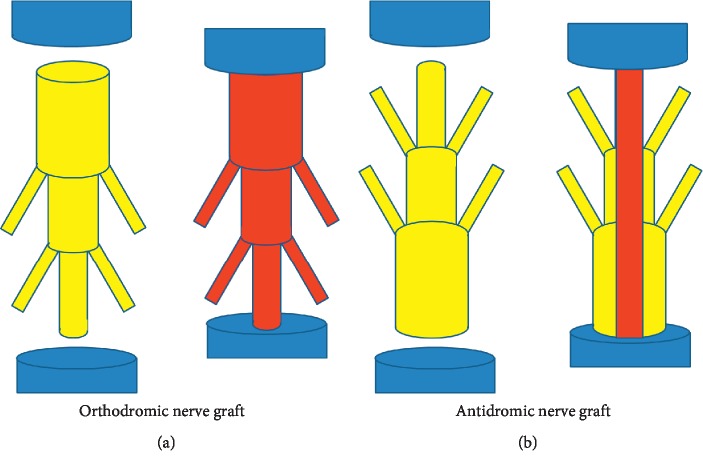
In an orthodromic nerve graft that includes nerve branches, regenerated axons that pass through the proximal repaired site may sprout into branches. However, in an antidromic nerve graft, because of the smaller cross-sectional area of the distal than the proximal end of the grafted nerve segment, fewer axons pass through the proximal repair site. Thus, the total number of axons passing through the respective repair sites in the two nerve grafts is the same.

There were no significant differences in the CMAP, nerve conduction velocity, or total axon count between the orthodromic and antidromic nerve graft groups in this study. Of the six studies included in a systematic review of the effect of autograft polarity on functional outcomes following nerve graft [4], four [5–7, 25] reported no difference and two [8, 20] significant differences between a normally oriented and a reversed graft. In 1988, Ansselin and Davey used a rodent sciatic nerve model to examine axonal regeneration following peripheral nerve graft according to graft polarity [20]. In the group that received a normally oriented graft, regenerating axons sprouted into branches instead of spanning the entire repair zone, which correlated with a decreased cross-sectional area of the distal nerve. The authors also noted that 12 months after the graft procedure, small branches persisted in 63% of the normally oriented grafts, which correlated with a smaller cross-sectional diameter than in rats that received a reversed graft [8]. However, the presence of many small branches in the grafted nerve segment implies a smaller cross-sectional area of the distal than the proximal end of the nerve segment (Figure 8). Therefore, there would be fewer regenerated axons and fewer axons passing through the proximal repair site in the reversed graft. Theoretically, the total number of axons that would be regenerated and pass through the distal repair site should be the same between orthodromic and antidromic nerve grafts.

Nerve grafting is usually indicated if *a* > 10% elongation of the nerve would be necessary to bridge the defect [26]. If the nerve is repaired under tension, the results of an interpositional nerve graft will be superior to those of the nerve repair [27] because axon sprouts more easily across two tension-free anastomotic sites than a single anastomosis site that is under tension [28]. Terzis et al. compared the results of reinnervation through nerve gaps sutured under tension or bridged with a nerve graft [29]. Regeneration through mildly stretched repair sites was equivalent to that obtained with a properly tailored graft, whereas excessively long grafts or moderately stretched repair sites led to poor results. In our study, the significant difference in outcomes between the nerve graft groups and the nerve repair group suggested that nerve repair under moderate tension is better than a nerve graft, regardless of the polarity.

## 5. Conclusion

In conclusion, there was no significant difference in the outcome of nerve regeneration between the orthodromic and antidromic nerve graft groups. However, the outcome of both nerve graft types was significantly poorer than that achieved with nerve repair. Therefore, direct nerve repair with moderate tension may be a more effective treatment than nerve graft. Further studies are needed to determine the tension affording the best possible outcome in nerve repair compared with a nerve graft performed without tension.

## Data Availability

The data used to support the findings of this study are included within the article.
